# An expression quantitative trait loci-guided co-expression analysis for constructing regulatory network using a rice recombinant inbred line population

**DOI:** 10.1093/jxb/ert464

**Published:** 2014-01-13

**Authors:** Jia Wang, Huihui Yu, Xiaoyu Weng, Weibo Xie, Caiguo Xu, Xianghua Li, Jinghua Xiao, Qifa Zhang

**Affiliations:** National Key Laboratory of Crop Genetic Improvement, National Center of Plant Gene Research, Huazhong Agricultural University, Wuhan 430070, PR China

**Keywords:** Gene expression, network construction, phenotype, quantitative genomics, regulation.

## Abstract

The ability to reveal the regulatory architecture of genes at the whole-genome level by constructing a regulatory network is critical for understanding the biological processes and developmental programmes of organisms. Here, we conducted an eQTL-guided function-related co-expression analysis to identify the putative regulators and construct gene regulatory network. We performed an eQTL analysis of 210 recombinant inbred lines (RILs) derived from a cross between two *indica* rice lines, Zhenshan 97 and Minghui 63, the parents of an elite hybrid, using data obtained by hybridizing RNA samples of flag leaves at the heading stage with Affymetrix whole-genome arrays. Making use of an ultrahigh-density single-nucleotide polymorphism bin map constructed by population sequencing, 13 647 eQTLs for 10 725 e-traits were detected, comprising 5079 *cis*-eQTLs (37.2%) and 8568 *trans*-eQTLs (62.8%). The analysis revealed 138 *trans*-eQTLs hotspots, each of which apparently regulates the expression variations of many genes. Co-expression analysis of functionally related genes within the framework of regulator–target relationships outlined by the eQTLs led to the identification of putative regulators in the system. The usefulness of the strategy was demonstrated with the genes known to be involved in flowering. We also applied this strategy to the analysis of QTLs for yield traits, which also suggested likely candidate genes. eQTL-guided co-expression analysis may provide a promising solution for outlining a framework for the complex regulatory network of an organism.

## Introduction

Expression of genes is co-ordinately regulated in space and time in an organism to deliver the gene products when needed, according to developmental programmes and in response to environmental cues (Schmid *et al.*, 2005; Wang *et al.*, 2010b). The ability to reveal the regulatory architecture of genes at the whole-genome scale by constructing a regulatory network is critical for understanding the biological processes and genome function of an organism (Davidson *et al.*, 2002; Bar-Joseph *et al.*, 2003; Shinozaki *et al.*, 2003).

In the past, technical approaches have been developed in two major ways to characterize gene expression regulation. The first is the detection technology of gene expression, ranging from traditional analysis of one gene at a time using Northern blotting, reverse transcription-PCR and real-time quantitative PCR (qPCR), to large-scale analysis of many genes, such as microarrays composed of all the annotated genes in an entire genome (Schena *et al.*, 1995; Tan *et al.*, 2003), and more recently by RNA sequencing (Wang *et al.*, 2009).

In parallel, there has also been the development of approaches to determine regulatory relationships of the genes. It is a common practice in the literature to identify the downstream targets of an altered gene using mutants or nearly isogenic lines (NILs) to analyse expression profiles for genes of interest, and then to expand the regulatory pathways stepwise using more mutants or NILs (Doi *et al.*, 2004; Espinosa-Soto *et al.*, 2004; Xue *et al.*, 2008). Another approach involves co-expression analysis by calculating correlations between genes based on large volumes of expression data, which can also provide useful information on how likely the gene functions are to be related (van Noort *et al.*, 2003; Zhang and Horvath, 2005; Wang *et al.*, 2010b). Expression quantitative trait loci (eQTLs) are genomic regions that regulate the gene expression variations in a population. Analysis of eQTLs that treats the expression values of the genes as a quantitative trait (e-trait) to perform QTL analysis using a genetically segregating population appears to be a useful strategy for suggesting regulatory relationships between genes (Gilad *et al.*, 2008; Kliebenstein, 2009). eQTL analysis can detect genetic elements that regulate the expression variation of the e-trait acting in *cis* if the QTL is located in the vicinity of an e-trait, or in *trans* if it is located in a distant position. Such analysis could suggest the existence of a potential regulator in a genomic region for a gene (Hansen *et al.*, 2008; Kliebenstein, 2009). Moreover, one regulator could be the target of another regulator. Thus, connecting such regulator–target relationships revealed by multiple e-traits and eQTLs of different layers would lead to a net-like structure, providing basic elements for the construction of a regulatory network. More recently, an approach combining eQTL with co-expression analysis was proposed to identify regulator candidates underlying eQTLs, which may greatly refine the resolution of the analysis (Terpstra *et al.*, 2010; Flassig *et al.*, 2013).

We attempted to construct a regulatory network adopting an eQTL-guided co-expression analysis strategy using flag leaf, a tissue that contributes significantly to grain yield as a primary source of carbohydrate production, from a population of 210 recombinant inbred lines (RILs) in rice. We showed that the analysis could identify candidate regulators and their targets down to the level of single genes. We verified the usefulness of this approach using genes in the flowering pathways. The results suggest that this analysis may provide a promising solution for outlining the framework for the complex regulatory network of an organism.

## Materials and methods

### Plant material

The population used in the study contained 210 RILs derived by single-seed descent from a cross between two *indica* rice lines, Zhenshan 97 and Minghui 63. The RILs and two parents were planted in a seed bed and transplanted to an experimental farm at Huazhong Agricultural University in the rice growing seasons in Wuhan, China. Ten seedlings were planted for each of the RILs in two different batches at 45 d apart as two biological replicates. Flag leaves from three random plants of each RIL per replicate were collected at the heading stage (day of the panicle emergence; Supplementary Fig. S1 available at *JXB* online) between 8:00 and 9:30 a.m.

RNA extraction, isolation, and Agilent Bioanalyzer quality testing were conducted for each of the two biological replicates. High-quality RNA samples of the two replicates were mixed (1:1) for cDNA synthesis, labelling, microarray hybridization and scanning. All processes were conducted by CapitalBio Corporation (Beijing, China).

NIL(mh7) and Zhenshan 97, which differ only by an introgressed genome segment, for the network verification were planted and the tissue samples collected in the same way as the RIL population.

### Microarray analysis

R platform (http://www.R-project.org) and ‘Affy’ packages from bioconductor (http://www.bioconductor.org) were applied to manage all the CEL files. Robust multiarray average (RMA) analysis was used for background correction and probe set expression value collection. Probe sets identified by MAS 5.0 algorithm as ‘P’ or ‘M’ for at least one-third of the 210 RILs were employed as e-traits for eQTL analysis.

### eQTL and phenotypic QTL (pQTL) analyses

eQTL and classical pQTL analyses were based on a genetic map consisting of 1619 recombinant bins constructed using high-quality single-nucleotide polymorphisms (SNPs) from resequencing of the 210 RILs (Yu *et al.*, 2011). The R/qtl function *cim* (Broman *et al.*, 2003) was employed for eQTL and pQTL mapping using a 10 cM scan window and five marker covariates (Potokina *et al.*, 2008; Yu *et al.*, 2011). The logarithm of the odds (LOD) threshold for eQTL identification was obtained based on a global permutation test that randomly selects 100 e-traits from all 21 929 e-traits to do 1000 permutations (West *et al.*, 2007; Wang *et al.*, 2010a). The eQTL additive effect and variation explained by the eQTL were determined using the linear QTL model described by Yu et al (2011). With the ultrahigh-density SNP bin map, the LOD thresholds of pQTL analysis for 10 yield-associated traits at *P*=0.05 ranged from 4.76 to 5.10 using 1000 permutation test for each phenotypic trait, with an average LOD value of 4.97. We used 5.0 as the threshold for pQTL identification.

### Real-time PCR analysis

RNA was isolated from NIL(mh7) and Zhenshan 97 rice flag leaves at the heading stage using TRIzol reagent (Invitrogen). First-strand cDNA was synthesized from DNase I-treated total RNA using Superscript III reverse transcriptase (Invitrogen) according to the manufacturer’s instructions. qPCR was performed with gene-specific primers (Supplementary Table S1 available at *JXB* online) in Applied Biosystems ViiA™ 7 using SYBR Premix Ex Taq (Takara) for six biological replicates of each sample. The program was 95 °C for 10 s, followed by 40 cycles of 95 °C for 5 s and 60 °C for 34 s, and relative expression levels were calculated by the 2^–ΔΔCT^ method (Livak and Schmittgen, 2001) compared with levels of expression of the ubiquitin gene.

### Accession codes

The National Center for Biotechnology Information Gene Expression Omnibus microarray data have been submitted under accession number GSE49020.

## Results

### Distribution of e-traits

In total, 44 029 (76.7%) of the 57 194 probe sets on the GeneChip Rice Genome Array each had a locus support in the Rice Genome Pseudomolecules (Release version MSU 6.1) representing 35 885 genes. In addition, 21 929 (49.8%) of the 44 029 probe sets corresponding to 17 934 genes were identified as ‘present’ in more than one-third of 210 RILs using the ‘mas5calls’ algorithm (Liu *et al.*, 2002) and were thus regarded as e-traits. We mapped the e-traits to the ultrahigh-density SNP bin map consisting of 1619 bins (Yu *et al.*, 2011). The distribution of e-traits was highly uneven across the genome (Supplementary Fig. S2 available at *JXB* online), and their numbers in the 1619 bins deviated significantly from the expectation based on physical sizes of the bins as shown by a χ^2^ test (χ^2^=47 264, *P*=2.2E–16) using the *chisq.test* function for a goodness-of-fit test. Further evaluation using the standardized residue (SR), which follows an asymptotical normal distribution, showed that the numbers of e-traits in 274 bins were significantly different from the expectations (Supplementary Table S2 available at *JXB* online). The density of the genes and e-traits in the centromeric and pericentromeric regions was lower than that in other chromosomal regions.

### Genome-wide eQTL mapping

**Fig. 1.**
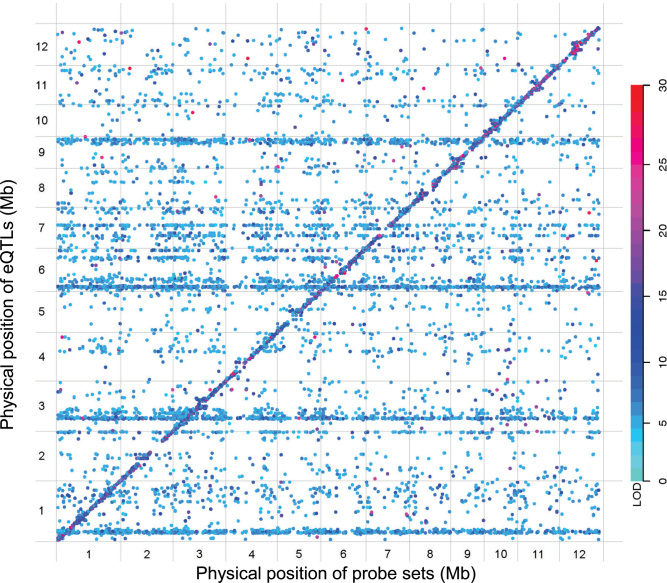
eQTLs identified using flag leaf tissue at the heading stage of RILs from a cross between Zhenshan 97 and Minghui 63. The positions of 13 647 eQTLs for 10 725 e-traits in the genome are shown. The *x*-axis shows the physical positions of expressed probe sets (e-traits) in the genome, and the *y*-axis the physical positions of eQTLs. Each dot represents an eQTL detected. The diagonal indicates possible *cis*-eQTLs, which are co-located with their corresponding probe sets. All the off-diagnal points indicate *trans*-eQTLs. The 12 rice chromosomes are separated by grey lines. The color key reflects the LOD scores (LOD scores >30 are set to 30). Most of the dots in higher scores are located in the diagonal representing *cis*-eQTLs. Multiple horizontal bands (*trans*-eQTL hotspots) are shown on four chromosomes (1, 3, 6, and 9), which suggests that transcript levels for many of the probe sets are associated with the polymorphisms in these regions. LOD scores >4.95 were adopted as the cut-off point for eQTLs.

These e-traits were subjected to eQTL analysis based on the ultrahigh-density SNP bin map using composite interval mapping (Zeng, 1993, 1994). We applied an LOD threshold of 4.95 (*P*=0.05), which was determined by a global permutation test, to identify eQTLs. An eQTL was regarded as *cis*-acting if the gene (e-trait) was within the 1.5 LOD-drop support interval (SI) of the corresponding eQTL, or as *trans*-acting if the gene (e-trait) was not located within the 1.5 LOD-drop SI (Keurentjes *et al.*, 2007). These processes eventually resulted in 13 647 eQTLs for 10 725 e-traits (Fig. 1 and Supplementary Table S3 available at *JXB* online), including 5079 *cis*-eQTLs (37.2%) and 8568 *trans*-eQTLs (62.8%) (Table 1). Only one eQTL was detected for 8258 (77.0%) of the 10 725 e-traits, and more than two eQTLs were detected for 407 (3.79%) of the e-traits. Most of the *cis-*eQTLs had greater LOD values and effects on gene expression than *trans*-eQTLs (Fig. 1 and Supplementary Fig. S3 available at *JXB* online). Overall, 69.2% of the *cis*-eQTLs each explained ≥20% of the expression variation for the corresponding e-traits, while only 8.79% of the *trans*-eQTLs could explain ≥20% of the expression variation for the corresponding e-traits.

**Table 1.** Statistics of the eQTLs on each chromosome

Chr.	eQTLs on chr.	*Cis-*eQTL	*Trans-*eQTL	Chr. size (Mb)	Expected^*a*^	SR^*b*^	Chr. size (cM)	Expected^*c*^	SR^*d*^	cM/Mb
1	2563	943	1620	43.3	1588	24.5	201	1684	21.4	4.6
2	929	463	466	35.9	1316	–10.7	175	1473	–14.2	4.9
3	1855	579	1276	36.4	1335	14.3	188	1574	7.1	5.2
4	706	379	327	35.3	1294	–16.4	127	1068	–11.1	3.6
5	608	373	235	29.9	1096	–14.7	116	974	–11.7	3.9
6	2453	410	2043	31.2	1144	38.7	144	1212	35.6	4.6
7	1135	290	845	29.7	1089	1.4	135	1137	–0.1	4.6
8	610	326	284	28.4	1041	–13.4	120	1010	–12.6	4.2
9	1093	356	737	23	843	8.6	107	900	6.4	4.7
10	509	332	177	23.1	847	–11.6	85	716	–7.1	3.7
11	739	380	359	28.5	1045	–9.5	117	980	–7.7	4.1
12	447	248	199	27.5	1008	–17.7	109	918	–15.5	4.0
	13647	5079	8568	372.2	13647		1625	13647		4.4

^*a*^Expected number of eQTLs based on chromosome size from physical map. χ^2^ = 3690.2 (*P* < 2.2e–16) for the test of goodness of fit between the observed and expected numbers of eQTLs on the 12 chromosomes.

^*b*^SR: for physical map, standardized residue [=(observed – expected)/√expected], which follows a normal distribution asymptotically. An absolute SR value >2.33 indicates statistical significance at *P*<0.01. A positive value indicates that the observed number is greater than expected.

^*c*^Expected number of eQTLs based on chromosome size from genetic map. χ^2^ = 2801.18 (*P* < 2.2e–16).

^*d*^SR: standardized residue for genetic map.

### *Trans*-eQTL hotspots

Four chromosomes (1, 3, 6, and 9) had higher numbers of eQTLs than expected based on both physical and genetic maps (Table 1), suggesting possible eQTL hotspots, as indicated by the horizontal bands (*trans*-eQTL hotspots) in Fig. 1. Comparison of observed and expected numbers of *trans*-eQTLs showed that *trans*-eQTLs detected in bins on some chromosomes (1, 3, 6, 7, and 9) were obviously more than the e-traits with *cis*-eQTLs (Supplementary Table S4 available at *JXB* online). We also performed a χ^2^ test for goodness of fit between observed and expected *trans*-eQTLs followed by calculating the SR, and a hypergeometric test for observed *trans*-eQTLs and e-traits with *cis*-eQTLs in the bins. It was found that the numbers of *trans*-eQTLs in 138 bins were significantly more than the expectations based on the sizes of the bins (*P*<0.01) and the numbers of e-traits with *cis*-eQTLs (*P*<0.01) (Supplementary Table S4 available at *JXB* online), indicating both *trans*-eQTL hotspots and coldspots. In particular, the top five bins of the *trans*-eQTL hotspots (Bin851 on chromosome 6; Bin857 on chromosome 6, Bin37 on chromosome 1, Bin 394 on chromosome 3, and Bin1012 on chromosome 7) ranging from 20 to 50kb harboured 439, 190, 169, 124, and 111 *trans*-eQTLs, respectively.

### Identifying candidate master regulators

**Fig. 2.**
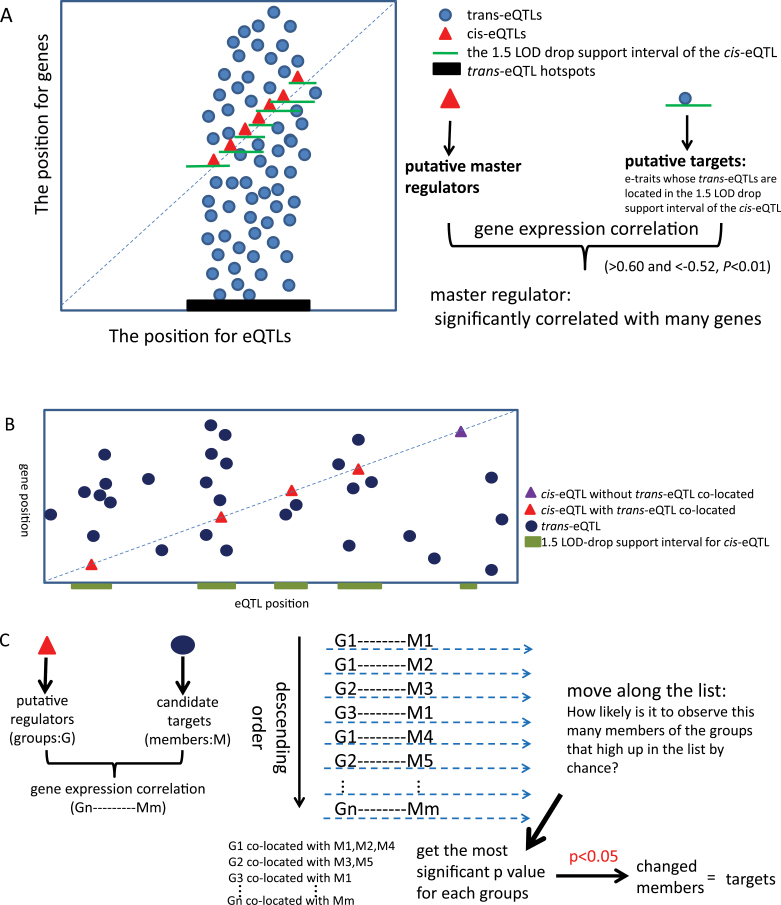
Schematic diagram of the procedure for identification of master regulators and construction of regulatory network for functionally related genes. (A) Identification of master regulators in the *trans*-eQTL hotspots by eQTL mapping. (B) eQTL map of functionally related genes. (C) The process of seeking master regulators and their targets according to the expression correlation between putative regulators and candidate targets for constructing a regulatory network (see main text for details).

A regulator at the high-node level in the network, or master regulator, frequently regulates many genes involved in multiple biological processes. We thus reasoned that: (1) such a regulator should correspond to a *trans*-eQTL hotspot, and (2) it should be co-expressed (positively or negatively) with the genes (targets) it regulates. To validate such an assertion and find candidate master regulators, we performed a co-expression analysis by calculating correlations between e-traits with *cis*-eQTLs and e-traits whose *trans*-eQTLs are located in the 1.5 LOD-drop SI of the *cis*-eQTLs (Fig. 2A). The threshold for significant correlations (0.60 and –0.52, *P*<0.01) was appraised based on 1 000 000 pair-wise correlations from two randomly selected groups each containing 1000 e-traits. Six genes seemed to be putative master regulators, as each was associated with more than 100 genes as the targets (Table 2), and other 27 genes obtained 20–100 candidate targets (Supplementary Table S5 available at *JXB* online). A gene ontology analysis based on biological processes of the associated targets for the six genes revealed that the targets for two of the putative regulators (Os.12583.1.S1_at annotated as expressed protein and Os.53449.1.A1_at annotated as OMP85 family protein) seemed to be highly enriched in certain functional categories (Supplementary Table S6 available at *JXB* online). No obvious enrichment was detected for the other four putative master regulators, indicating that these putative master regulators might be involved in diverse regulatory pathways.

**Table 2.** Information on the six putative master regulators genes, associated with more than 100 genes as targetsThe candidate regulators were suggested to be the gene with *cis*-eQTLs that co-mapped with *trans*-eQTLs of many co-expressed genes (*P*<0.01).

Regulator	*N* ^*a*^	Bin	Chr.	LOD^*b*^	Inf. Mb^*c*^	Sup. Mb^*d*^	Var^*e*^	Annotation^*f*^
Os.3539.1.S1_at	412	Bin851	6	5.61	2.89	4.50	7.45%	Vesicle-associated membrane protein, putative, expressed
Os.12583.1.S1_at	213	Bin389	3	14.77	8.66	9.53	22.27%	Outer-membrane protein, OMP85 family, putative, expressed
Os.53449.1.A1_at	204	Bin395	3	6.80	8.75	10.04	11.39%	Expressed protein
Os.9960.1.S1_at	145	Bin857	6	23.56	3.29	4.02	35.33%	WD-40 repeat family protein, putative, expressed
Os.16410.1.S1_s_at	127	Bin853	6	4.96	2.89	4.50	4.61%	BSD domain containing protein, expressed
Os.27513.1.A1_a_at	103	Bin36	1	5.45	4.75	6.04	5.17%	Phosphoenolpyruvate carboxylase, putative, expressed

^*a*^ *N*, the number of co-expression probe sets with *trans*-eQTLs located in the 1.5 LOD-drop interval of corresponding *cis*-eQTL of the e-trait.

^*b*^LOD, the LOD value for the *cis*-eQTL for the regulator.

^*c*^Inf.Mb, the inferior position for the 1.5 LOD-drop interval of *cis*-eQTLs.

^*d*^Sup.Mb, the superior position for the 1.5 LOD-drop interval of *cis*-eQTLs.

^*e*^Var, the expression variation explained by the *cis*-eQTL for the regulator.

^*f*^Annotation, the gene annotation for the regulator.

### Network construction for functionally related genes

Gene regulatory networks usually regulate the expression of arrays of genes in specific spatial and temporal patterns. To explore the feasibility of using the expression data to identify regulatory networks, we performed co-expression analysis of genes known to be involved in the long-day flowering pathway. Many studies have revealed that, as a short-day plant, rice flowering is regulated by both a short-day activation pathway and a long-day suppression pathway (Hayama *et al.*, 2003). Our RIL populations were planted in the rice growing season (May–September) in Wuhan, China, in which the natural day length is 13–14.5h (nearly a long day).

An iterative group analysis (iGA) algorithm (Breitling *et al.*, 2004; Keurentjes *et al.*, 2007) was used to analyse the e-trait expression profile to find master regulators of the flowering genes, based on the assumption that a concerted expression variation was controlled by the same regulator and that the functions of the genes were physiologically relevant. In this process (Fig. 2B, C), a putative local regulator gene that has a *cis*-eQTL co-located with a *trans*-eQTL was assigned a group name, and an e-trait with a *trans*-eQTL that co-mapped with the group was assigned as a member of the group. We first sorted the putative targets (members) according to the expression correlations with potential regulators (groups) in descending order, and moved along the list counting the targets of each candidate regulator. When a new target was encountered each time, we inquired: How likely is it that we would observe this many targets of the regulator that high up in the list by chance? For each group, we defined the probability of change (PC) value, which indicated how likely a regulator with *cis*-eQTL is to show a strong correlation with several members with *trans*-eQTLs of a selected group of genes. A regulatory group with a significant PC value (*P*<0.05) would be regarded as a regulator controlling the members contributing to this PC value.

We illustrated this procedure using the flowering regulation genes as an example. We identified 577 unique loci annotated as known or predicted genes in the category GO:0009908 (flower development), as well as ones known to be involved in flower development and the flowering time pathway from the literature (Supplementary Table S7 available at *JXB* online). There were 280 eQTLs associated with 177 of the 577 unique probe sets including 109 *cis*-eQTLs and 171 *trans*-eQTLs (Supplementary Table S8 available at *JXB* online). Sixty-one of the 109 genes with *cis*-eQTLs had a SI overlapping the SI of *trans*-eQTLs. We calculated expression correlations between the 61 genes (potential regulators) and 171 genes with *trans*-eQTLs (potential targets), which amounted to 7808 gene pairs when disregarding the genes that had both *cis-* and *trans-*eQTLs (Supplementary Table S9 available at *JXB* online). The correlation coefficients were rank ordered. iGA (Breitling *et al.*, 2004) was applied to select the most significant groups and the significant change targets contributing to PC value, which indicate how likely a regulator with *cis*-eQTL is to observe a strong correlation with several members with *trans*-eQTLs of a selected group of genes.

*Hd3a* and *RFT1*, essential for flowering in rice, share 91% identity in their deduced amino acid sequences and are located only 11.5kb apart on chromosome 6 (Komiya *et al.*, 2008). A *cis*-eQTL was detected for *RFT1*, but the expression variation of *Hd3a* mapped no significant locus. To avoid the obscuring effect of sequence identity between the genes, the expression levels of *RFT1* and *Hd3a* were recalculated using a subset of four probes selected from the 11 probes in the chip for each gene that had more than five SNPs between *RFT1* and *Hd3a*. With this analysis, the effect of the *cis*-eQTL for *RFT1* was greatly elevated with the LOD increasing from 7.33 to 76.02 (Supplementary Table S10 and Supplementary Fig. S4 available at *JXB* online), and the amount of variation explained from 9.9 to 12.3%. A *trans*-eQTL for *Hd3a* (LOD=5.2, *R* ^2^=9.2%), associated with Bin1006 (chromosome 7), emerged from this analysis (Supplementary Table S10 and Supplementary Fig. S5 available at *JXB* online). At the *cis*-eQTL for *RFT1* on chromosome 6, the Minghui 63 allele had a positive effect on *RFT1* expression. However, *RFT1* also had a *trans*-eQTL on chromosome 1, at which the Zhenshan 97 allele had a positive effect on *RFT1* expression. Consequently, the expression levels of *RFT1* did not seem to be very different in the two parents (Supplementary Fig. S6 available at *JXB* online). In contrast, the two parents showed large difference in the expression level of *Hd3a*, apparently due to the *trans*-eQTL on chromosome 7.

**Fig. 3.**
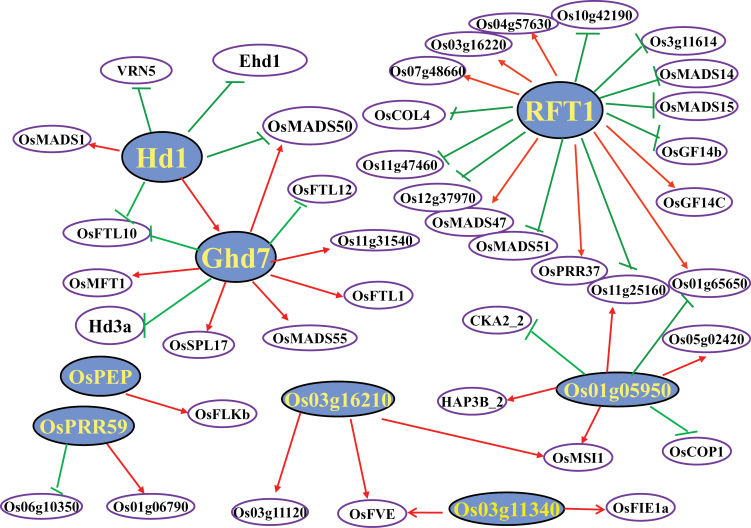
The regulatory network of genes involved in flower development and the flowering time pathway. Genes in yellow are putative regulators, while other genes connected to them in black are the candidate targets. Arrows indicate positive regulation and bars indicate negative regulation.

Eight obvious regulatory groups were identified (*P*<0.05) (Table 3) based on the iGA analysis, and the targets for each regulator are shown in Fig. 3, with the expression correlations displayed in Supplementary Table S11 available at *JXB* online. *Ghd7*, *Hd1*, and *RFT1* were identified as master regulators in this analysis, which were the *trans*-eQTLs for several flowering-related genes. The *trans*-eQTL for *Hd3a* appeared to be *Ghd7* (cor=–0.30, *P*=8.63E–6). Several *trans*-eQTLs for flowering-related genes seemed to be *RFT1*, of which *OsMADS14* and *OsMADS15* were identified as targets of *RFT1* in a previous work (Komiya *et al.*, 2008, 2009). Meanwhile, *OsGF14b* and *OsGF14c*, two 14-3-3 proteins, act as intracellular receptors for rice *Hd3a* florigen (Taoka *et al.*, 2011), and were identified as the targets of *RFT1* in our results. This is in agreement with the conclusion that *RFT1* was the major regulator for long-day flowering (Komiya *et al.*, 2009). *Ehd1* and *OsMADS50*, confirmed as upstream regulators of *RFT1/Hd3a*, both had *trans*-eQTLs co-located with *Hd1* on chromosome 6. Highly significant negative expression correlations of *Hd1* with *Ehd1* (cor=–0.54, *P*=4.06E–17) and *OsMADS50* (cor=–0.27, *P*=7.51E–05) in 210 RILs indicated that they both were downregulated by *Hd1*, suggesting *Hd1* as a regulator of *Ehd1* and *OsMADS50*. Others have reported that *Hd1* and *Ehd1* are independent in flowering regulation (Doi *et al.*, 2004; Takahashi *et al.*, 2009), and a more recent study showed that *Ehd1* appears to be negatively regulated and *Ghd7* was positively regulated by *Hd1* under long-day conditions (Song *et al.*, 2012), which seems to substantiate our network. We obtained the 2kb genomic sequence upstream of the ATG site of *Ehd1* and *OsMADS50*, and found no difference between Zhenshan 97 and Minghui 63, suggesting that this 2kb sequence may not be the cause of the expression difference. In contrast, *OsMADS50* was positively regulated by *Ghd7* (cor=0.25, *P*=2.05E–4) with a *trans*-eQTL (LOD=14.1, *R* ^2^=20%) in the genomic position of *Ghd7*.

**Table 3.** The eight regulatory groups identified in the analysis

Group^*a*^	Members^*b*^	Number changed^*c*^	*P* value changed^*d*^	Gene	MUS6.1
Os.15230.1.S1_at	24	19	1.14E–05	*RFT1*	LOC_Os06g06300
Os.10204.1.S1_at	10	7	1.08E–04	*Os01g09590*	LOC_Os01g09590
Os.12795.1.S1_at	2	2	7.68E–03	*OsPRR59*	LOC_Os11g05930
OsAffx.28467.1.S1_at	11	11	0.016	*Ghd7*	LOC_Os07g15770
Os.12674.1.S1_s_at	2	2	0.022	*Os03g11340*	LOC_Os03g11340
Os.1189.1.S1_at	6	6	0.032	*Hd1*	LOC_Os06g16370
Os.30077.2.S1_at	5	3	0.033	*Os03g16210*	LOC_Os03g16210
Os.2720.1.S1_at	3	1	0.042	*OsPEP*	LOC_Os10g41440

^*a*^Group, All local regulatory genes were classified as group names.

^*b*^Members, the number of e-traits with *trans*-eQTLs co-mapped with the corresponding group.

^*c*^Number changed, the number of candidate targets that may be controlled by the regulator.

^*d*^ *P* value changed, the probability of change, indicating the likelihood of observing the members of the group that high up in the list by chance.

**Fig. 4.**
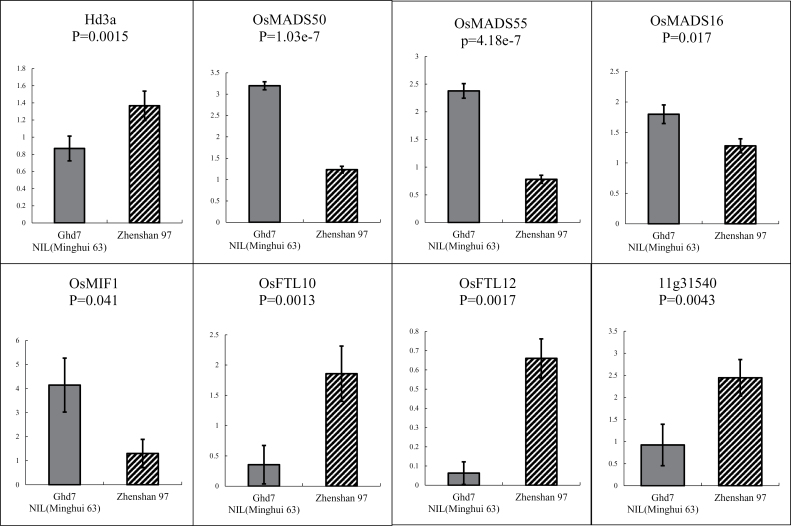
qPCR verification for the expression abundance of candidate targets in NIL(mh7) and Zhenshan 97. Flag leaves at the heading date were used for the analysis. The *y*-axis represents relative expression levels. Means ±standard error are based on six biological replicates.

To confirm the network, we checked the transcript abundance of the candidate downstream targets for modulator of *Ghd7* using flag leaves at heading date from Zhenshan 97 and NIL (mh7), which have an identical genetic background except for the introgressed segment (Xue *et al.*, 2008). The qPCR results showed that the transcript levels of *OsFTL10*, *OsFTL12*, and *Hd3a* were lower in NIL(mh7) than in Zhenshan 97, while the reverse was the case for *OsMFT1*, *OsMADS50*, *OsMADS55*, and *OsMADS16* (Fig. 4). This was consistent with the result presented in the network analysis (Supplementary Table S11 available at *JXB* online). However, the situation for *11g31540* was different from the analysis of the population data (Fig. 3). Moreover, *OsSPL17* and *OsFTL1*, putative targets of *Ghd7* that were identified with very low correlations (0.19, *P*=0.007; 0.24, *P*=4.8E–4), did not show significantly different gene expression between NIL(mh7) and Zhenshan 97. These discrepancies may be due to differences in the genetic material (RILs vs NILs) and/or environmental factors, as the *cis*-eQTL for *Ghd7* could not fully explain the expression variation of the gene in the population (LOD=67.6, *R* ^2^=68.5%).

### Application of cis-eQTLs for gene discovery of phenotypic traits

Genetic variations for gene expression and phenotypic quantitative traits are mostly the results of DNA sequence polymorphisms. Combining pQTL mapping with eQTL analysis may facilitate investigation of the molecular regulatory networks underlying quantitative traits. A gene with *cis*-eQTLs usually has functional polymorphisms in promoter and/or gene-coding regions, which may cause variations in gene expression levels and phenotypic effects. Indeed, phenotypic variations in a number of QTLs for phenotypic traits result from gene expression variation due to sequence polymorphisms (Chu *et al.*, 2006; Weng *et al.*, 2008; Li *et al.*, 2011). Flag leaves at the heading stage, the tissue used in this analysis, have a crucial role for rice yield. We thus used data for yield and yield-related traits for QTL mapping to identify candidate genes controlling the variation of the traits. With the ultrahigh-density SNP bin map, the LOD thresholds at *P*=0.05 ranged from 4.76 to 5.10 for 1000 permutation test, with an average LOD value of 4.97, and we selected 5.0 as the threshold for pQTLs (Supplementary Table S12 available at *JXB* online). We then calculated correlations between phenotypic trait scores and gene expression levels of e-traits with *cis*-eQTLs with SIs overlapping the corresponding pQTLs. The genes with significant correlations with the trait scores are shown for each trait in Supplementary Table S13 (available at *JXB* online). Interestingly, a pleiotropic QTL controlling the number of grains per panicle, plant height, heading date, yield per plant, and flag leaf width was shown to be associated with Bin1006 on chromosome 7. Three genes (*LOC_Os07g16030*, *LOC_Os07g15670*, and *LOC_Os07g15770*) with larger-effect *cis*-eQTLs were regarded as putative candidate genes, and exhibited significant correlations with the phenotypes of the four traits (Supplementary Table S14 available at *JXB* online). The cloned gene *Ghd7* (LOC_Os07g15770) has been shown to be a pleiotropic QTL for number of grains per panicle, plant height, heading date, and yield per plant (Xue *et al.*, 2008), and Zhenshan 97 lacked the *Ghd7* locus while Minghui 63 had a functional allele. This confirmed a *cis*-eQTL with a remarkable effect on the expression level of *Ghd7* (LOD= 67.6, *R* ^2^=68.5%).

## Discussion

Construction of a regulatory network is the basis for depicting regulation of gene expression at the whole-genome level, which is fundamental for an understanding of biological processes and the functions of both individual genes and the genome. A classical gene expression network is constructed based on one gene perturbation at a time, making use of genetic materials such as mutants and NILs. Although such analysis could provide reliable information on the immediate downstream target(s) of the perturbed gene, it may not be able to provide useful information for regulators and/or targets that are in more distant positions or upstream of the perturbed gene in the network. eQTL mapping seeks to understand the genetic basis of gene expression variation and identify causal relationships between gene expression and genetic variation by analysing multifactorial natural perturbations (Jansen and Nap, 2001; Logsdon and Mezey, 2010). However, like pQTLs, an eQTL corresponds to a genomic region of variable size, usually ranging from hundreds of kilobases to megabases, which still requires huge efforts to identify possible candidate genes.

In this study, we have presented a simple, yet effective method for gene regulatory network reconstruction using an eQTL-guided function-related co-expression analysis. We combined data for gene expression from a rice RIL population, gene functional annotation of prior knowledge and iGA with the co-expression level to identify candidate regulators. Such analysis is based on the hypothesis that a regulator in a high node of the network regulates many genes and would show *trans*-acting effects in the form of *trans*-eQTLs for many e-traits that show a correlated expression profile at the population level, and the targets and regulator would be involved in the same biological process.

We used genes involved in the flowering regulation as an example to demonstrate the utility of the method in constructing a gene regulatory network, and identified and validated with high confidence a number of elements and the pathways known to be involved in flowering regulation. Our results demonstrated that the strategy adopted here could precisely identify a putative regulator down to the single gene, representing a great improvement compared with ordinary eQTL analysis, which detected the *trans*-eQTL as an interval harbouring many genes. Compared with co-regulation analysis by simple correlations, the strategy here was able to provide a causal relationship between the co-expressed genes, which also represents a major improvement. In addition, the analysis revealed other genes in the network that have not been identified previously as elements in flowering regulation. Whether they really represent genes in the system that are yet to be discovered remains to be confirmed, but these may be used as clues for future experiments.

We further expanded such eQTL-guided co-expression analysis to the identification of candidate genes for pQTLs. The results also demonstrated the promise of this approach, which may lead to the identification of candidate genes controlling quantitative traits.

Another point to be discussed is that the tissue used in this study was flag leaf at the heading stage, at which time the plant is already flowering, well after flowering induction. This raises a question about the functions of these genes and the regulatory network, suggesting that they may also have functions in other biological processes besides flowering regulation. In fact, it has been shown that flowering-related genes often exhibit pleiotropic effects that cannot be explained by the extended length of the vegetative stage alone (Yan *et al.*, 2011, 2013). For example, *Ghd7* affects grain number, plant height, and heading date under long-day conditions (Xue *et al.*, 2008), and was also reported as controlling flag leaf area (Tan *et al.*, 2012). Combination of *Hd1* and *Ehd1* in rice can reduce the number of primary branches in a panicle, resulting in smaller spikelet numbers per panicle (Endo-Higashi and Izawa, 2011). Pleiotropic effects of ﬂowering-related genes have also been reported in other plants. *Arabidopsis LHP1* controls developmental pathways involved in organ and cell size (Gaudin *et al.*, 2001). The potato tuberization transitions are controlled by two different FT-like paralogues (*StSP3D* and *StSP6A*) that respond to independent environmental cues (Navarro *et al.*, 2011). Another observation is that flowering-related genes are not only expressed in the panicle, but also in the leaf, sheath, stem, hull, shoot, seedling, and even roots (Supplementary Fig. S7 available at *JXB* online) (Wang *et al.*, 2010b). All this evidence suggests that these firmly regarded ‘flowering genes’ have more functions than just flowering induction. However, whether the compositions and architectures of the regulatory networks in terms of the genes and pathways are the same in different processes remain to be determined.

Gene expression is a dynamic process depending on development stages, and different tissues or cells, as well as genetic backgrounds and growth environments (Jiao *et al.*, 2009; Wang *et al.*, 2010b). Thus, the results and interpretations of the eQTL analysis are also valid in terms of the tissues from which the RNA samples were collected. Whether the regulatory network and/or co-expression relationships between the genes identified in a single tissue study are tissue specific, or are to some extent also valid in other tissues, remains to be investigated.

It should also be noted that the outcome of the eQTL detection is critically dependent on the genetic composition of the mapping population defined by the polymorphisms between the parents. For example, Komiya *et al.* (2009) confirmed that the *OsMADS50*–*Ehd1*–*RFT1* pathway is involved in floral activation under long-day conditions. However, no *cis*-eQTL was detected for either *OsMADS50* or *Ehd1*, probably because of no sequence difference in the promoter regions of these genes between the parents of our population. Consequently, neither *OsMADS50* and *Ehd1* was identified as a *trans*-eQTL for other e-traits, and hence were not suggested as regulators by the eQTL analysis. Clearly, the parental genotypes pose a limiting factor for the extent to which the components in the regulatory network can be discovered in the analysis. Multiple populations of diverse parents may be used to provide complementary information for the network construction.

A further challenge is to identify the *cis*-acting element in each case that regulates the expression variation of the e-trait, or the causal agent of the *cis*-eQTL. The definition of *cis*-effect in eQTL analyses is somewhat arbitrary, varying from 5Mb (Morley *et al.*, 2004) to 10kb of the gene (Yvert *et al.*, 2003), or a 1.5 LOD-drop SI of the QTL (Keurentjes *et al.*, 2007). In principle, a *cis*-eQTL should be caused by polymorphisms within a neighbouring range of the gene, including polymorphisms within the gene itself that alter the *cis*-acting elements or sites for mRNA stability, or polymorphisms between parents in the promoter region (Heap *et al.*, 2009; Smith *et al.*, 2011). The above definition does not exclude the possibility that the regulatory element may act *in trans*, as in the case of the *AMN1* gene in *Saccharomyces cerevisiae* that was found to be a local but *trans*-acting through feedback regulation due to a coding polymorphism in its transcript (Ronald *et al.*, 2005). In any case, it is essential to find sequence polymorphisms between the parents and associate such polymorphisms with the expression level variation of the gene for identifying the *cis*-acting elements of the e-traits.

The large number of regulators and the regulatory network identified in the study only provide a starting point for understanding the system. Although we have illustrated that some are supported by results from previous studies, huge effort is needed experimentally to confirm any of the master regulators and the regulatory network.

## Supplementary data

Supplementary data is available at *JXB* online.

Supplementary Fig. S1. An example of a flag leaf harvested at the heading stage.

Supplementary Fig. S2. The distribution of e-traits and probe sets with locus support in the genome.

Supplementary Fig. S3. Distribution of *R* ^2^ values for *cis*-eQTLs and *trans*-eQTLs individually.

Supplementary Fig. S4. LOD curve for expression variation that was calculated with the full set of probe sets and modified probe sets of *RFT1* and *Hd3a* on chromosome 6.

Supplementary Fig. S5. LOD curve for expression variation, which was calculated with the full set of probe sets and modified probe sets of *RFT1* and *Hd3a* on chromosome 7.

Supplementary Fig. S6. Gene expression levels for *RFT1* and *Hd3a* in Zhenshan 97 and Minghui 63 of three biological replicates.

Supplementary Fig. S7. Heatmap of the global development stage of the flowering-related genes identified in our network.

Supplementary Table S1. Primers used in qPCR analysis for the candidate targets expression in NIL(mh7) and Zhenshan 97.

Supplementary Table S2. Numbers of observed and expected e-traits in 1619 bins.

Supplementary Table S3. Details of 13 647 significant gene expression quantitative trait loci (eQTLs) (*P*≥0.05), affecting the expression of 10 725 rice unique probe sets.

Supplementary Table S4. Information on observed *trans*-eQTLs, expected *trans*-eQTLs, and e-traits with *cis*-QTLs in 1619 bins.

Supplementary Table S5. The significant master candidate regulators.

Supplementary Table S6. The enrichment gene ontology (GO) terms for significantly associated targets for six master regulators.

Supplementary Table S7. Information on 577 unique loci involved in flowering time and development.

Supplementary Table S8. *Cis* and *trans*-eQTL identification of flowering-associated genes.

Supplementary Table S9. Total number of unique correlation pairs: 7808 for 61 candidate regulators and 171 potential targets.

Supplementary Table S10. Gene expression for *RFT1* and *Hd3a* was recalculated with the full set of probe sets and modified probe sets with probes with less than five SNPs removed between *RFT1* and *Hd3a*.

Supplementary Table S11. Expression correlation for regulator and corresponding targets.

Supplementary Table S12. The statistics of 25 pQTLs identified using the ultrahigh-density SNP bin map.

Supplementary Table S13. The most significant candidate genes for each trait.

Supplementary Table S14. Correlation between phenotypic traits and e-traits with *cis*-eQTL in the SI of QTL controlling corresponding traits.

Supplementary Data

## Abbreviations:

eQTL: expression quantitative trait locus
iGA: iterative group analysis
LOD: logarithm of the odds
NIL: or nearly isogenic line
PC: probability of change
qPCR: real-time quantitative PCR
pQTL: phenotypic quantitative trait locus
RIL: recombinant inbred line
SI: support interval
SNP: single-nucleotide polymorphisms
SR: standardized residue.

## References

[CIT0001] Bar-Joseph Z Gerber GK Lee TI 2003 Computational discovery of gene modules and regulatory networks. Nature Biotechnology 21, 1337–1342 10.1038/nbt890 14555958

[CIT0002] Breitling R Amtmann A Herzyk P 2004 Iterative Group Analysis (iGA): a simple tool to enhance sensitivity and facilitate interpretation of microarray experiments. BMC Bioinformatics 5, 34 15050037 10.1186/1471-2105-5-34 PMC403636

[CIT0003] Broman KW Wu H Sen S Churchill GA 2003 R/qtl: QTL mapping in experimental crosses. Bioinformatics 19, 889–890 12724300 10.1093/bioinformatics/btg112

[CIT0004] Chu Z Yuan M Yao J 2006 Promoter mutations of an essential gene for pollen development result in disease resistance in rice. Genes & Development 20, 1250–1255 16648463 10.1101/gad.1416306 PMC1472899

[CIT0005] Davidson EH Rast JP Oliveri P 2002 A genomic regulatory network for development. Science 295, 1669–1678 11872831 10.1126/science.1069883

[CIT0006] Doi K Izawa T Fuse T Yamanouchi U Kubo T Shimatani Z Yano M Yoshimura A 2004 Ehd1, a B-type response regulator in rice, confers short-day promotion of flowering and controls FT-like gene expression independently of Hd1. Genes & Development 18, 926–936 15078816 10.1101/gad.1189604 PMC395851

[CIT0007] Endo-Higashi N Izawa T 2011 Flowering time genes *Heading date 1* and *Early heading date 1* together control panicle development in rice. Plant and Cell Physiology 52, 1083–1094 21565907 10.1093/pcp/pcr059 PMC3110884

[CIT0008] Espinosa-Soto C Padilla-Longoria P Alvarez-Buylla ER 2004 A gene regulatory network model for cell-fate determination during *Arabidopsis thaliana* flower development that is robust and recovers experimental gene expression profiles. Plant Cell Online 16, 2923–2939 10.1105/tpc.104.021725 PMC527189 15486106

[CIT0009] Flassig RJ Heise S Sundmacher K Klamt S 2013 An effective framework for reconstructing gene regulatory networks from genetical genomics data. Bioinformatics 29, 246–254 23175757 10.1093/bioinformatics/bts679

[CIT0010] Gaudin V Libault M Pouteau S Juul T Zhao G Lefebvre D Grandjean O 2001 Mutations in *LIKE HETEROCHROMATIN PROTEIN 1* affect flowering time and plant architecture in Arabidopsis. Development 128, 4847–4858 11731464 10.1242/dev.128.23.4847

[CIT0011] Gilad Y Rifkin SA Pritchard JK 2008 Revealing the architecture of gene regulation: the promise of eQTL studies. Trends in Genetics 24, 408–415 18597885 10.1016/j.tig.2008.06.001 PMC2583071

[CIT0012] Hansen BG Halkier BA Kliebenstein DJ 2008 Identifying the molecular basis of QTLs: eQTLs add a new dimension. Trends in Plant Science 13, 72–77 18262820 10.1016/j.tplants.2007.11.008

[CIT0013] Hayama R Yokoi S Tamaki S Yano M Shimamoto K 2003 Adaptation of photoperiodic control pathways produces short-day flowering in rice. Nature 422, 719–722 12700762 10.1038/nature01549

[CIT0014] Heap GA Trynka G Jansen RC Bruinenberg M Swertz MA Dinesen LC Hunt KA Wijmenga C Vanheel DA Franke L 2009 Complex nature of SNP genotype effects on gene expression in primary human leucocytes. BMC Medical Genomics 2, 1 19128478 10.1186/1755-8794-2-1 PMC2628677

[CIT0015] Jansen RC Nap JP 2001 Genetical genomics: the added value from segregation. Trends in Genetics 17, 388–391 11418218 10.1016/s0168-9525(01)02310-1

[CIT0016] Jiao Y Tausta SL Gandotra N 2009 A transcriptome atlas of rice cell types uncovers cellular, functional and developmental hierarchies. Nature Genetics 41, 258–263 19122662 10.1038/ng.282

[CIT0017] Keurentjes JJ Fu J Terpstra IR 2007 Regulatory network construction in Arabidopsis by using genome-wide gene expression quantitative trait loci. Proceedings of the National Academy of Sciences, USA 104, 1708–1713 10.1073/pnas.0610429104 PMC1785256 17237218

[CIT0018] Kliebenstein D 2009 Quantitative genomics: analyzing intraspecific variation using global gene expression polymorphisms or eQTLs. Annual Review of Plant Biology 60, 93–114 10.1146/annurev.arplant.043008.092114 19012536

[CIT0019] Komiya R Ikegami A Tamaki S Yokoi S Shimamoto K 2008 Hd3a and RFT1 are essential for flowering in rice. Development 135, 767–774 18223202 10.1242/dev.008631

[CIT0020] Komiya R Yokoi S Shimamoto K 2009 A gene network for long-day flowering activates RFT1 encoding a mobile flowering signal in rice. Development 136, 3443–3450 19762423 10.1242/dev.040170

[CIT0021] Li Y Fan C Xing Y 2011 Natural variation in GS5 plays an important role in regulating grain size and yield in rice. Nature Genetics 43, 1266–1269 22019783 10.1038/ng.977

[CIT0022] Liu WM Mei R Di X 2002 Analysis of high density expression microarrays with signed-rank call algorithms. Bioinformatics 18, 1593–1599 12490443 10.1093/bioinformatics/18.12.1593

[CIT0023] Livak KJ Schmittgen TD 2001 Analysis of relative gene expression data using real-time quantitative PCR and the 2^–ΔΔCT^ method. Methods 25, 402–408 11846609 10.1006/meth.2001.1262

[CIT0024] Logsdon BA Mezey J 2010 Gene expression network reconstruction by convex feature selection when incorporating genetic perturbations. PLoS Computational Biology 6, e1001014 21152011 10.1371/journal.pcbi.1001014 PMC2996324

[CIT0025] Morley M Molony CM Weber TM Devlin JL Ewens KG Spielman RS Cheung VG 2004 Genetic analysis of genome-wide variation in human gene expression. Nature 430, 743–747 15269782 10.1038/nature02797 PMC2966974

[CIT0026] Navarro C Abelenda JA Cruz-Oro E Cuellar CA Tamaki S Silva J Shimamoto K Prat S 2011 Control of flowering and storage organ formation in potato by FLOWERING LOCUS T. Nature 478, 119–122 21947007 10.1038/nature10431

[CIT0027] Potokina E Druka A Luo Z Wise R Waugh R Kearsey M 2008 Gene expression quantitative trait locus analysis of 16 000 barley genes reveals a complex pattern of genome-wide transcriptional regulation. The Plant Journal 53, 90–101 17944808 10.1111/j.1365-313X.2007.03315.x

[CIT0028] Ronald J Brem RB Whittle J Kruglyak L 2005 Local regulatory variation in Saccharomyces cerevisiae. Plos Genetics 1, e25 16121257 10.1371/journal.pgen.0010025 PMC1189075

[CIT0029] Schena M Shalon D Davis RW Brown PO 1995 Quantitative monitoring of gene expression patterns with a complementary DNA microarray. Science 270, 467–470 7569999 10.1126/science.270.5235.467

[CIT0030] Schmid M Davison TS Henz SR Pape UJ Demar M Vingron M Scholkopf B Weigel D Lohmann JU 2005 A gene expression map of *Arabidopsis thaliana* development. Nature Genetics 37, 501–506 15806101 10.1038/ng1543

[CIT0031] Shinozaki K Yamaguchi-Shinozaki K Seki M 2003 Regulatory network of gene expression in the drought and cold stress responses. Current Opinion in Plant Biology 6, 410–417 12972040 10.1016/s1369-5266(03)00092-x

[CIT0032] Smith RM Alachkar H Papp AC Wang D Mash DC Wang JC Bierut LJ Sadee W 2011 Nicotinic α5 receptor subunit mRNA expression is associated with distant 5′ upstream polymorphisms. European Journal of Human Genetics 19, 76–83 20700147 10.1038/ejhg.2010.120 PMC2995013

[CIT0033] Song Y Gao Z Luan W 2012 Interaction between temperature and photoperiod in regulation of flowering time in rice. Science China Life Sciences 55, 241–249 22527521 10.1007/s11427-012-4300-4

[CIT0034] Takahashi Y Teshima KM Yokoi S Innan H Shimamoto K 2009 Variations in Hd1 proteins, Hd3a promoters, and Ehd1 expression levels contribute to diversity of flowering time in cultivated rice. Proceedings of the National Academy of Sciences, USA 106, 4555–4560 10.1073/pnas.0812092106 PMC2647979 19246394

[CIT0035] Tan C Weng XY Yan WH Bai XF Xing YZ 2012 Ghd7, a pleiotropic gene controlling flag leaf area in rice. Yi Chuan 34, 901–906 22805217 10.3724/sp.j.1005.2012.00901

[CIT0036] Tan PK Downey TJ Spitznagel Jr EL Xu P Fu D Dimitrov DS Lempicki RA Raaka BM Cam MC 2003 Evaluation of gene expression measurements from commercial microarray platforms. Nucleic Acids Research 31, 5676–5684 14500831 10.1093/nar/gkg763 PMC206463

[CIT0037] Taoka K Ohki I Tsuji H 2011 14–3-3 proteins act as intracellular receptors for rice Hd3a florigen. Nature 476, 332–335 21804566 10.1038/nature10272

[CIT0038] Terpstra IR Snoek LB Keurentjes JJ Peeters AJ van den Ackerveken G 2010 Regulatory network identification by genetical genomics: signaling downstream of the Arabidopsis receptor-like kinase ERECTA. Plant Physiology 154, 1067–1078 20833726 10.1104/pp.110.159996 PMC2971588

[CIT0039] van Noort V Snel B Huynen MA 2003 Predicting gene function by conserved co-expression. Trends in Genetics 19, 238–242 12711213 10.1016/S0168-9525(03)00056-8

[CIT0040] Wang J Yu H Xie W Xing Y Yu S Xu C Li X Xiao J Zhang Q 2010a A global analysis of QTLs for expression variations in rice shoots at the early seedling stage. The Plant Journal 63, 1063–1074 20626655 10.1111/j.1365-313X.2010.04303.x

[CIT0041] Wang L Xie W Chen Y 2010b A dynamic gene expression atlas covering the entire life cycle of rice. The Plant Journal 61, 752–766 20003165 10.1111/j.1365-313X.2009.04100.x

[CIT0042] Wang Z Gerstein M Snyder M 2009 RNA-Seq: a revolutionary tool for transcriptomics. Nature Reviews Genetics 10, 57–63 10.1038/nrg2484 PMC2949280 19015660

[CIT0043] Weng J Gu S Wan X 2008 Isolation and initial characterization of GW5, a major QTL associated with rice grain width and weight. Cell Research 18, 1199–1209 19015668 10.1038/cr.2008.307

[CIT0044] West MA Kim K Kliebenstein DJ van Leeuwen H Michelmore RW Doerge RW St Clair DA 2007 Global eQTL mapping reveals the complex genetic architecture of transcript-level variation in Arabidopsis. Genetics 175, 1441–1450 17179097 10.1534/genetics.106.064972 PMC1840073

[CIT0045] Xue W Xing Y Weng X 2008 Natural variation in Ghd7 is an important regulator of heading date and yield potential in rice. Nature Genetics 40, 761–767 18454147 10.1038/ng.143

[CIT0046] Yan W Liu H Zhou X 2013 Natural variation in Ghd7.1 plays an important role in grain yield and adaptation in rice. Cell Research 23, 969–971 23507971 10.1038/cr.2013.43 PMC3698629

[CIT0047] Yan W Wang P Chen H 2011 A major QTL, Ghd8, plays pleiotropic roles in regulating grain productivity, plant height, and heading date in rice. Molecular Plant 4, 319–330 21148627 10.1093/mp/ssq070

[CIT0048] Yu H Xie W Wang J Xing Y Xu C Li X Xiao J Zhang Q 2011 Gains in QTL detection using an ultra-high density SNP map based on population sequencing relative to traditional RFLP/SSR markers. PLoS ONE 6, e17595 21390234 10.1371/journal.pone.0017595 PMC3048400

[CIT0049] Yvert G Brem RB Whittle J Akey JM Foss E Smith EN Mackelprang R Kruglyak L 2003 Trans-acting regulatory variation in *Saccharomyces cerevisiae* and the role of transcription factors. Nature Genetics 35, 57–64 12897782 10.1038/ng1222

[CIT0050] Zeng ZB 1993 Theoretical basis for separation of multiple linked gene effects in mapping quantitative trait loci. Proceedings of the National Academy of Sciences, USA 90, 10972–10976 10.1073/pnas.90.23.10972 PMC47903 8248199

[CIT0051] Zeng ZB 1994 Precision mapping of quantitative trait loci. Genetics 136, 1457–1468 8013918 10.1093/genetics/136.4.1457 PMC1205924

[CIT0052] Zhang B Horvath S 2005 A general framework for weighted gene co-expression network analysis. Statistical Applications in Genetics and Molecular Biology 4, 1128 10.2202/1544-6115.1128 16646834

