# Supplementary material for: An expression quantitative trait loci-guided co-expression analysis for constructing regulatory network using a rice recombinant inbred line population
Source: J Exp Bot. 2014 Jan 13;65(4):1069–79. doi: 10.1093/jxb/ert464 (PMC3935569; doi:10.1093/jxb/ert464)
Supplement: Supplementary Data [file supp_65_4_1069__index.html]

An expression quantitative trait loci-guided co-expression analysis for constructing regulatory network using a rice recombinant inbred line population — An expression quantitative trait loci-guided co-expression analysis for constructing regulatory network using a rice recombinant inbred line population — Supplementary Data 

# An expression quantitative trait loci-guided co-expression analysis for constructing regulatory network using a rice recombinant inbred line population

## Supplementary Data

Data files

**Files in this Data Supplement:**

- Supplementary Data - Supplementary Data
- Supplementary Data - Supplementary Data
