# Supplementary material for: An expression quantitative trait loci-guided co-expression analysis for constructing regulatory network using a rice recombinant inbred line population
Source: J Exp Bot. 2014 Jan 13;65(4):1069–79. doi: 10.1093/jxb/ert464 (PMC3935569; doi:10.1093/jxb/ert464)
Supplement: Supplementary Data [file supp_ert464_jexbot108209_file001.pdf]

An eQTL guided co-expression analysis for constructing regulatory network using a rice RIL population

Jia Wang, Huihui Yu, Xiaoyu Weng, Weibo Xie, Caiguo Xu, Xianghua Li, Jinghua Xiao & Qifa Zhang*

Supplementary Figure S1

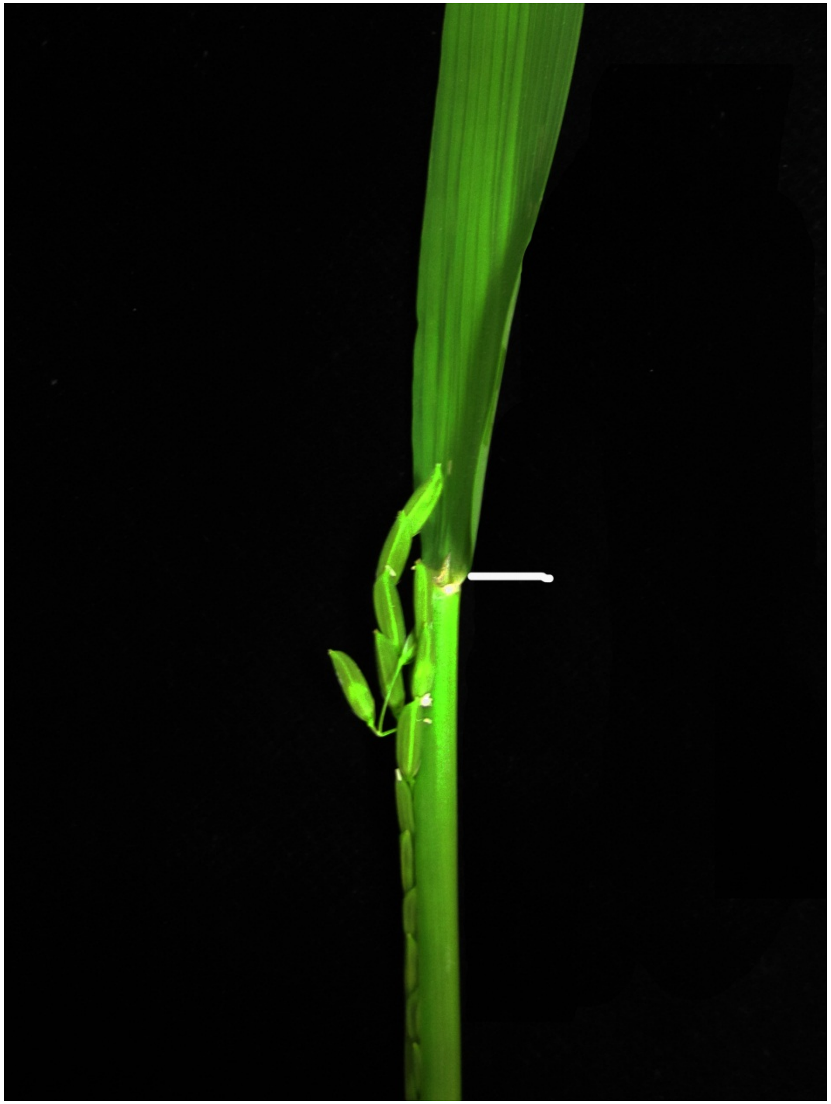

Supplementary Figure S1. An example for the flag leaf we harvested at heading stage. The white bar means the sampling site and the whole leaf were used in the research

Supplementary Figure S2

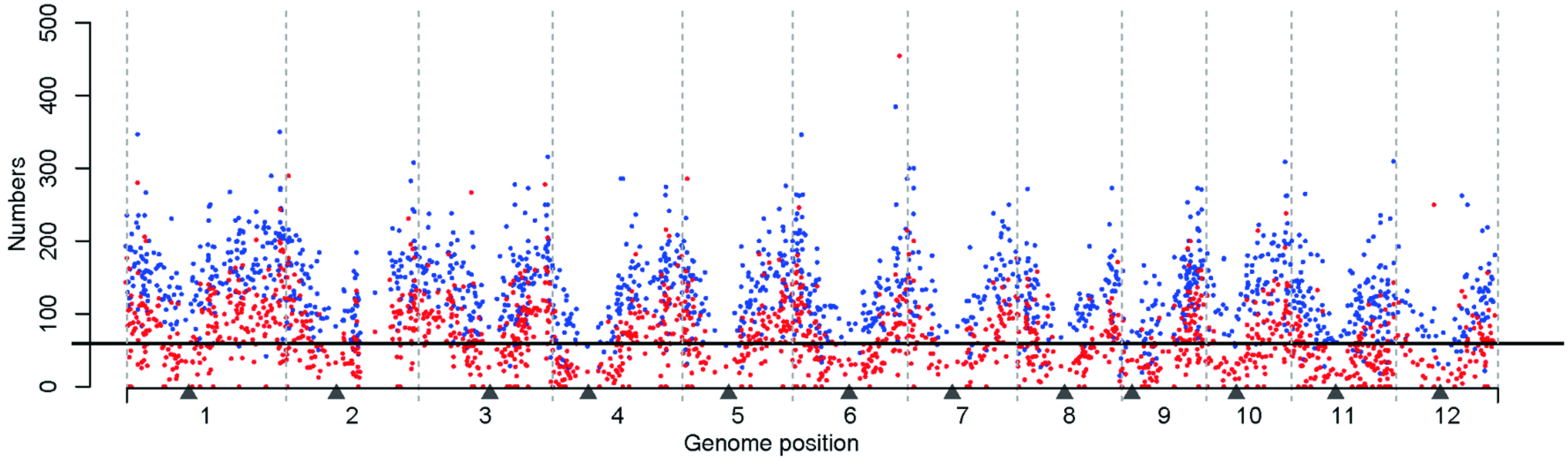

Supplementary Figure S2**.** The distribution of e-traits and probesets with locus-support in the genome.

The x-axis indicates genomic locations by chromosomal order, and the y-axis indicates the density of e-traits in the genomic region (measured by the numbers of e-traits/Mb and locus-supported probesets/Mb in each bin). The red and blue points indicate e-traits and locus-supported probesets respectively. The grey triangle shows the position of centromere. The horizontal bar means the number of all e-traits/genome size.

Supplementary Figure S3

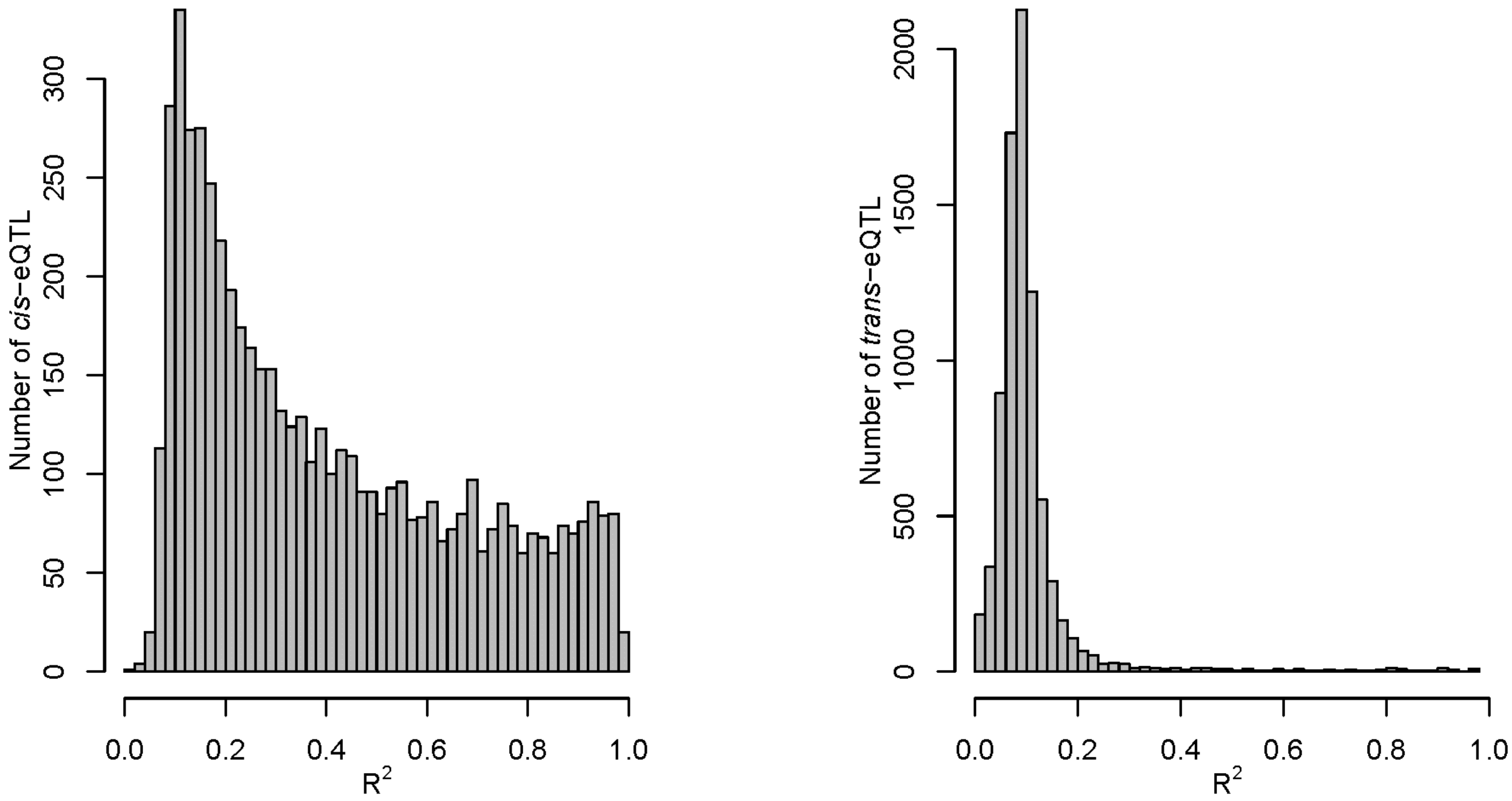

Supplementary Figure S2. Distribution of R^2 values for *cis*-eQTLs and *trans*-eQTLs individually.

R^2 values represent expression variation for the corresponding e-traits explained by the eQTLs.

(a) The histogram of R^2 values for *cis*-eQTLs.

(b) The histogram of R^2 values for *trans*-eQTLs.

Supplementary Figure S4

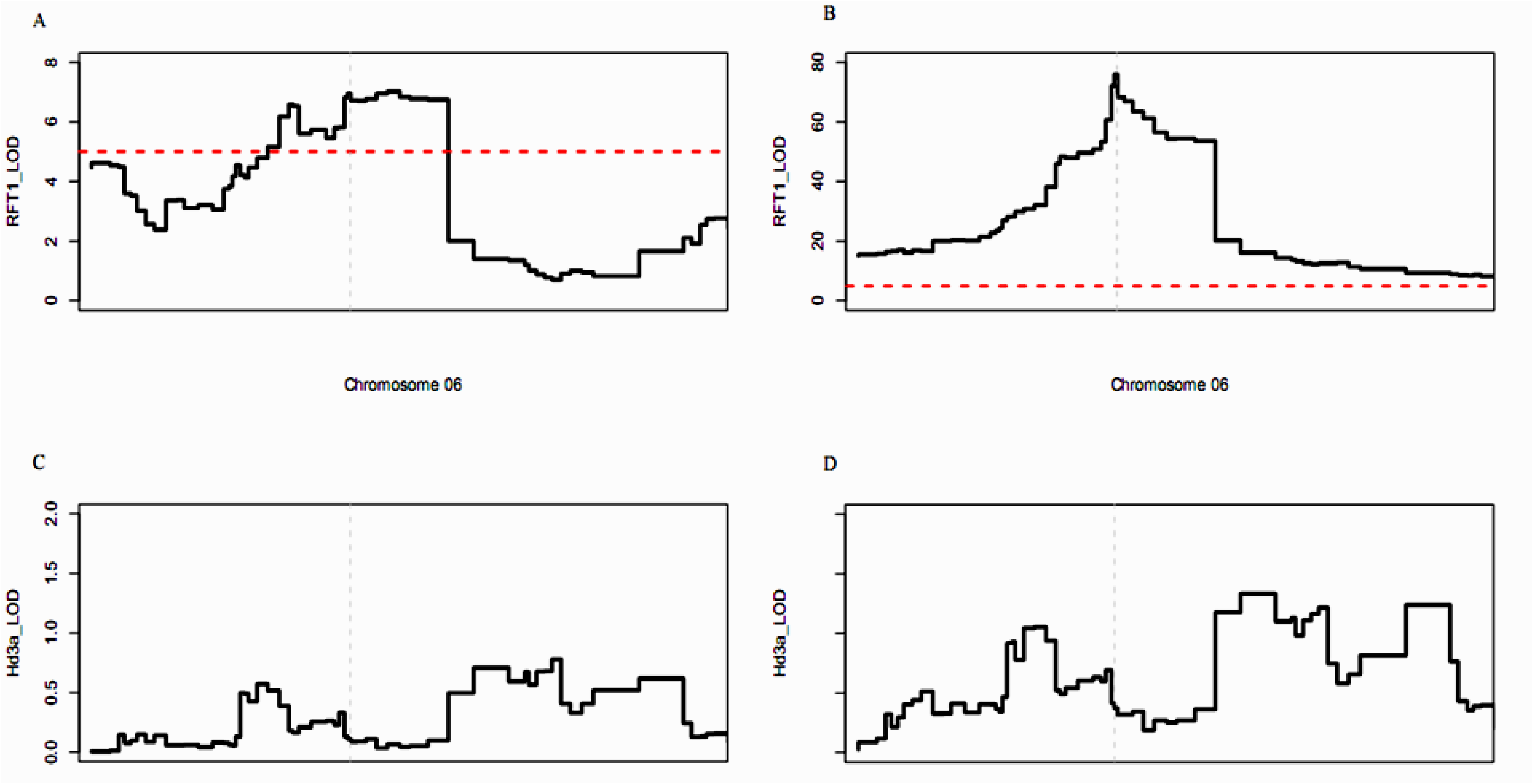

Supplementary Figure S3. LOD curve for expression variation which was calculated with the full set of probesets and modified probesets of *RFT1* and *Hd3a* on chr06.

A, LOD curve for expression variation with the full set of probesets of *RFT1* on chr06.

B, modified probesets of *RFT1*.

C, the full set of probesets of *Hd3a*.

D, modified probesets of *Hd3a*.

Supplementary Figure S5

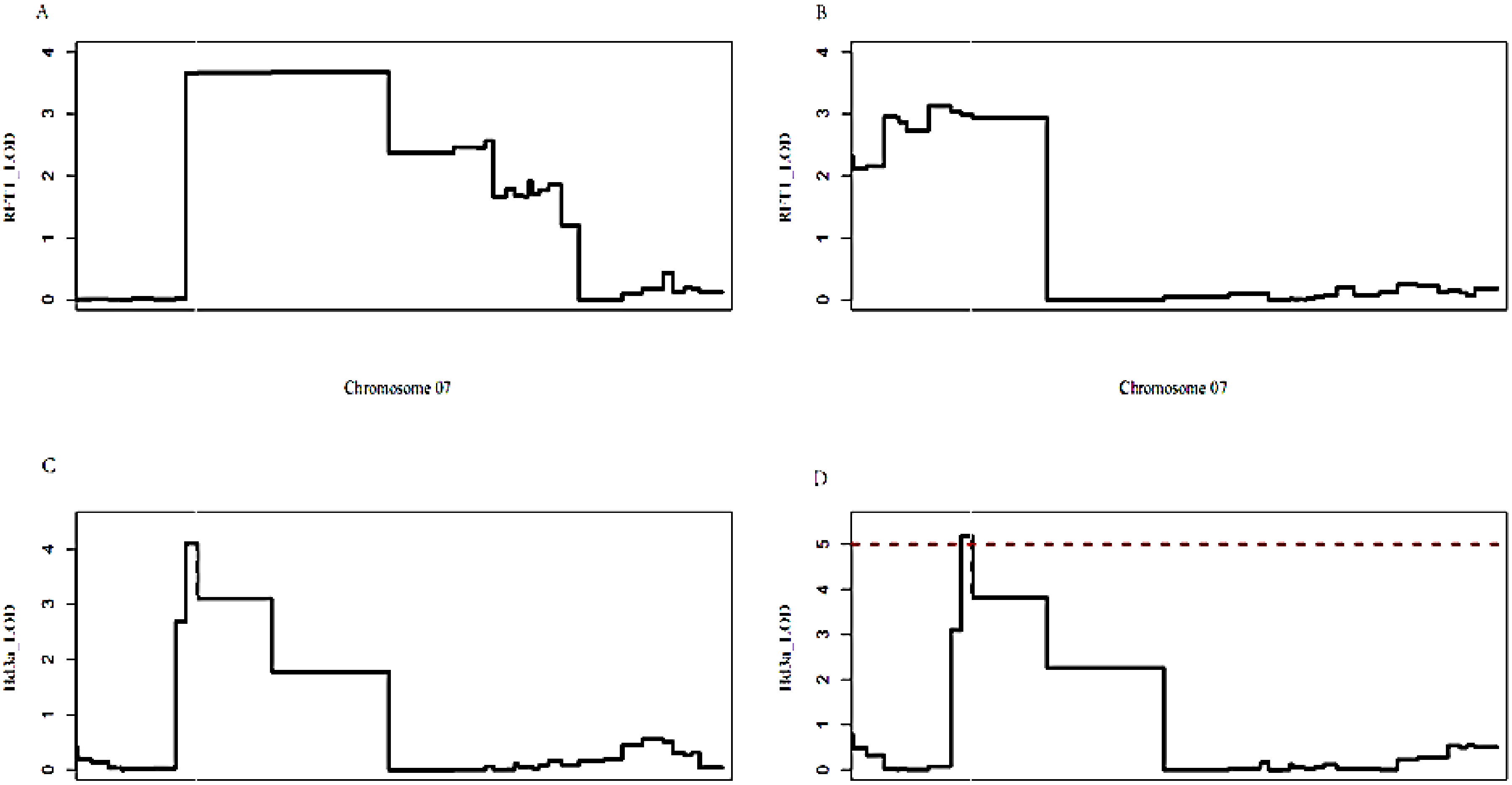

Supplementary Figure S4. LOD curve for expression variation which was calculated with the full set of probesets and modified probesets of *RFT1* and *Hd3a* on chr07.

A, LOD curve for expression variation with the full set of probesets of *RFT1* on chr07.

B, modified probesets of *RFT1*.

C, the full set of probesets of *Hd3a*.

D, modified probesets of *Hd3a*.

Supplementary Figure S6

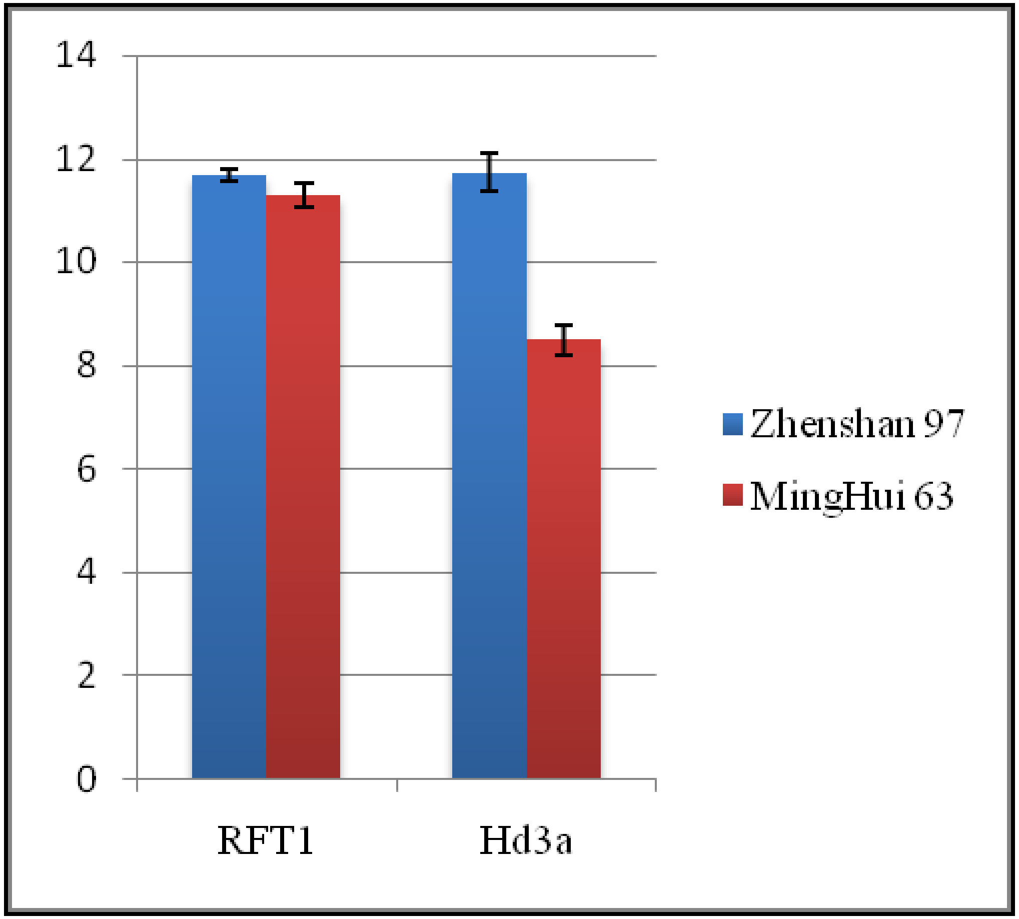

Supplementary Figure S5. The gene expression levels for *RFT1* and *Hd3a* in Zhenshan97 and Minghui 63 of three biological replicates.

Supplementary Figure S7

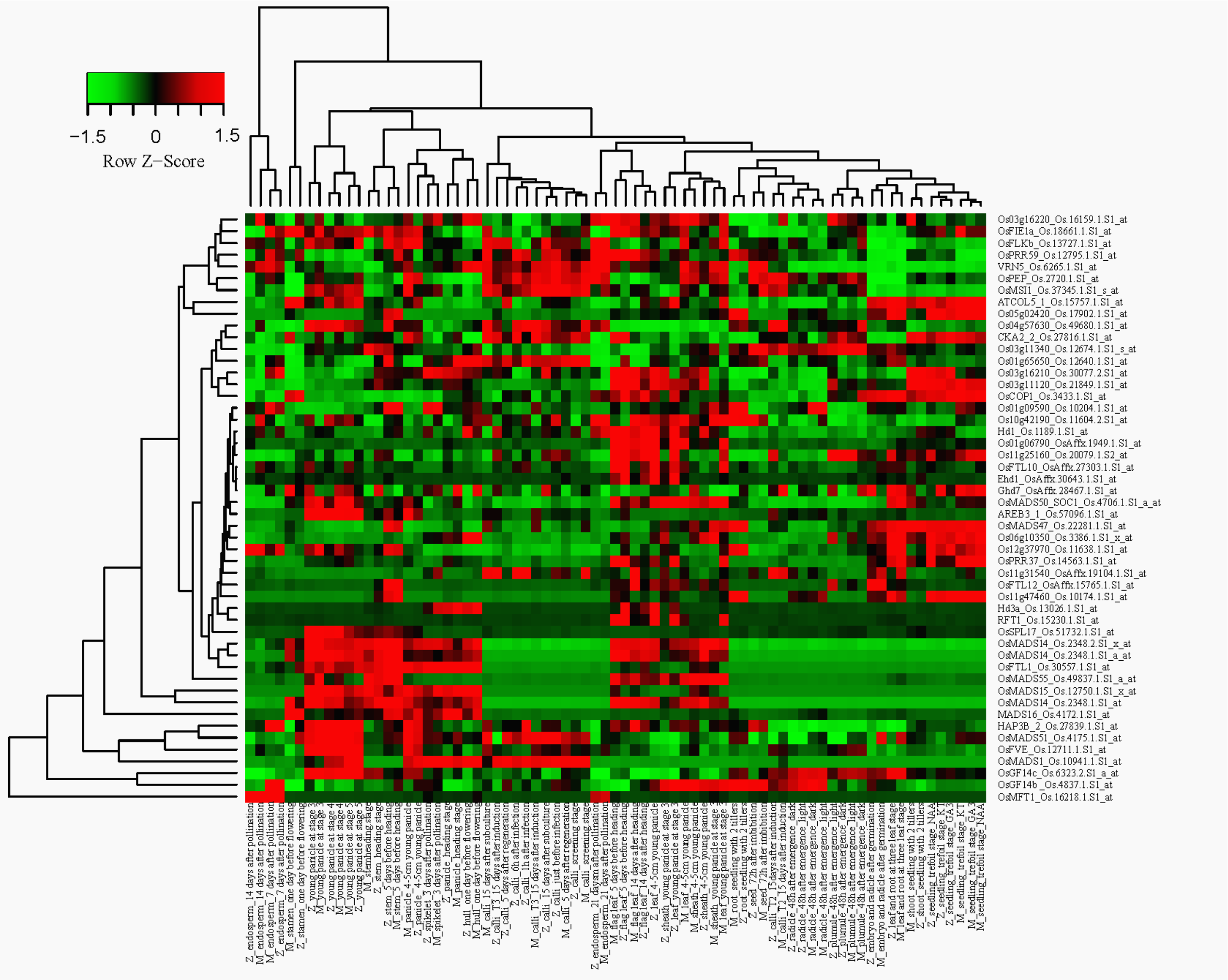

Supplementary Figure S6. The heatmap of global development stage of the flowering related genes identified in our network.

The columns indicate the tissue and development in Zhenshan 97 and Minghui 63. Z means Zhenshan 97; M means Minghui 63. The rows shows the genes identified in our flowering related network.
